# Vitamin D as a Modifiable Risk Factor for Juvenile Idiopathic Arthritis: A Systematic Review and Meta-analysis of Observational Studies Comparing Baseline Vitamin D in Children with JIA to Individuals Without

**DOI:** 10.1093/nutrit/nuae148

**Published:** 2024-10-25

**Authors:** Kathleen Zang, Resham Bhatia, Elizabeth Xue, Kalia J Bennett, Katherine H Luo, Monali S Malvankar-Mehta

**Affiliations:** Department of Epidemiology and Biostatistics, Schulich School of Medicine and Dentistry, The University of Western Ontario, London, ON, Canada; Department of Epidemiology and Biostatistics, Schulich School of Medicine and Dentistry, The University of Western Ontario, London, ON, Canada; Department of Epidemiology and Biostatistics, Schulich School of Medicine and Dentistry, The University of Western Ontario, London, ON, Canada; Department of Epidemiology and Biostatistics, Schulich School of Medicine and Dentistry, The University of Western Ontario, London, ON, Canada; Department of Epidemiology and Biostatistics, Schulich School of Medicine and Dentistry, The University of Western Ontario, London, ON, Canada; Department of Epidemiology and Biostatistics, Schulich School of Medicine and Dentistry, The University of Western Ontario, London, ON, Canada; Department of Ophthalmology, Schulich School of Medicine and Dentistry, The University of Western Ontario, London, ON, Canada

**Keywords:** juvenile idiopathic arthritis (JIA), vitamin D, pediatric, systematic review, meta-analysis

## Abstract

**Context:**

The varying interactions contributing to the development of juvenile idiopathic arthritis (JIA) drive the struggle to understand its etiology. Among the environmental risk factors, vitamin D has been posited to have a component in disease progression, acting as an inflammatory mediator.

**Objective:**

To investigate the correlation between serum 25-hydroxyvitamin D [25(OH)D] levels, indicative of vitamin D, among patients diagnosed with JIA compared with control participants. The aim was to elucidate potential therapeutic implications of vitamin D in the management of JIA.

**Data Sources:**

A systematic search of 6 electronic databases (MEDLINE, Embase, Scopus, CINAHL, Web of Science, and Cochrane Library) was performed until February 2023. Inclusion criteria required participants to be <16 years old (either clinically diagnosed with JIA or a matched control participant), with vitamin D levels measured through serum laboratory methods. Exclusion criteria omitted studies in which participants used vitamin D supplementation or medications affecting vitamin D levels without corresponding statistical analyses on their association with vitamin D levels.

**Data Extraction:**

Each article was reviewed by at least 2 independent reviewers to assess eligibility for analysis.

**Data Analysis:**

Data were qualitatively analyzed to compare means of serum 25(OH)D levels (ng/mL) between patients with JIA and control participants, followed by a meta-analysis to obtain effect size.

**Results:**

Ten eligible studies were included qualitatively, and eight were included in the meta-analysis. Seven studies found a statistically significant difference in vitamin D levels between control participants and patients with JIA, with five of these reporting a lower mean vitamin D level in patients with JIA. A random-effects model using standardized mean difference found a statistically significant difference in vitamin D levels between control participants and patients with JIA (–0.49; 95% CI, –0.92 to –0.06).

**Conclusions:**

The findings from the analysis indicate vitamin D levels were lower in patients with JIA as compared with healthy control participants at baseline. It is recommended that research into vitamin D supplementation and JIA should be conducted.

## INTRODUCTION

Juvenile idiopathic arthritis (JIA), formerly called juvenile rheumatoid arthritis and juvenile chronic arthritis, is the onset of arthritis from unknown causes before the age of 16 years. Its time frame is defined by the persistence of symptoms over 6 weeks. Although there may be a lack of cognizance among the lay public of JIA, approximately 3 million children and adolescents suffer from it globally.^1^ Given the extensive nature of the disease, it can be a cause of considerable disability and poor quality of life, demonstrated by functional outcome assessments that generally place emphasis on years lost to children. Additionally, it imposes large societal burdens in the form of economic costs and strain on guardians and caregivers.^2^ Over the past decade, functional outcomes of JIA have seen improvement; however, long-term prognosis is still imperfect because of gaps in understanding of the exact etiology of the disease.^3^ Across JIA’s 6 subtypes, common symptoms include joint pain, swelling, decreased range of motion, and stiffness.^4^

Recent studies have suggested that JIA arises from various immune mechanisms, depending on its subtype.^3^ In general, JIA is a disease that arises from autoimmune inflammatory processes.^5^ There has been some research into the influence of genetics and immunology on the development of JIA that, although not yet proven, remains promising for future courses of study. Due to the idiopathic nature of the disease, there has been increasingly more research focused on determining its etiology and pathogenesis. The heterogeneity of the disease, exemplified by its many subtypes, suggests both environmental and genetic factors play a role in its genesis.^5^ Subtypes of JIA are identified by the International League of Rheumatology as oligoarticular, seropositive polyarticular, seronegative polyarticular, systemic-onset, enthesitis-related, juvenile psoriatic, and undifferentiated.^6^

One avenue of research into environmental factors has focused on the role of vitamin D deficiency and the subsequent development of JIA. Vitamin D is known to have anti-inflammatory and bone health–promoting properties, and deficient levels have been suspected to be a possible modifiable risk factor^7^ to target to reduce suppression of immune responses and inflammation in those with JIA.^8^ Vitamin D incorporated in diets or synthesized in the skin from sun exposure requires successive hydroxylations in the liver and kidney to create its biologically active form.^9^ The resulting metabolite, 25-hydroxyvitamin D [25(OH)D], circulates in the blood and is often used to identify a patient’s vitamin D levels. In general, a 25(OH)D level < 20 ng/mL is considered deficient and 21-29 ng/mL is categorized as insufficient; the ideal vitamin D level in children is >30 ng/mL.^8^ There has been some evidence that vitamin D deficiency is associated with indicators of greater inflammation,^9^ in addition to having a higher prevalence in those with JIA.^10^

The current body of evidence lends itself well to the rationale of a systematic review. The last systematic review on this topic was published by Nisar et al^11^ in 2013, which focused on vitamin D supplementation, and Finch et al^12^ published an additional scoping review in 2018. These past reviews were limited to a few papers with conclusions calling for more standardized measurements of vitamin D levels. An updated systematic review of relevant literature is required to further understand juvenile vitamin D levels and the effect of vitamin D as an inflammatory mediator, and to explore the role of deficiency and optimal vitamin D status in children.

Our objective for this systematic review was to compare vitamin D levels (measured as serum 25[OH]D levels, considered the gold standard barometer) between patients with JIA and control participants to identify possible associations between the disease and vitamin D status. This analysis aims to elucidate the potential therapeutic implications of vitamin D in the management and development of JIA.

## METHODS

### Inclusion and Exclusion Criteria

This review adhered to the Preferred Items for Systematic Reviews and Meta-Analyses (PRISMA) guidelines.^13^ Criteria for eligible studies were based on the PICOS (population, intervention, comparison, outcomes, and study design) format (Table 1). All randomized experiments and nonrandomized and observational studies were included (ie, no restrictions on the types of studies). The criteria for literature inclusion were as follows: (1) the text was available in English within a peer-reviewed journal; (2) the full text was available; (3) participants in studies were humans <16 years of age; (4) JIA was clinically diagnosed; (5) vitamin D levels were either clinically or laboratory measured (eg, enzyme-linked immunosorbent assays); and (6) studies of interest either examined the implications of vitamin D levels in children with JIA compared with children without or compared vitamin D levels among JIA subgroups. To manage language bias, the search strategy was executed both with and without the English filter. The resulting comparison yielded few additional results, indicating minimal impact on the overall search outcome.

**Table 1.** PICOS Criteria for Inclusion of Studies

Parameter	Inclusion criteria	Exclusion criteria
Population	Pediatric patients (children) <16 years old when study first began; all sexes and races	
Intervention	Vitamin D levels, measured in preexisting blood or serum levels	Studies including vitamin D supplementation or other medication influencing vitamin D levels, without a statistical analysis reporting their association with each other; non–serum level measurements (genetic indicators, not 25-hydroxyvitamin D specific)
Comparison	Children without juvenile idiopathic arthritis; juvenile idiopathic arthritis subgroups	
Outcomes	Juvenile idiopathic arthritis	Those without a clinical diagnosis
Study design	Cross-sectional studies; randomized controlled trials; nonrandomized controlled trials; cohort studies; case-control studies	Studies without full report available

The criteria for literature exclusion were as follows: (1) if a single-case report or case-series design was used; (2) if a report was a review, a commentary, or a meta-analysis; (3) if a full description of the research design was not provided or available, or if there were incomplete trials (no results); or (4) if the participants used vitamin D supplementation or other medications that influence vitamin D levels (eg, corticosteroids), but no statistical analyses were conducted to comment on supplementation or medications’ association with vitamin D levels.

### Search Methods for Identification of Studies

The following bibliographic databases were searched for eligible studies until February 2, 2023: MEDLINE (Ovid), Embase, Scopus, Cumulative Index to Nursing and Allied Health Literature (CINAHL), Web of Science, and Cochrane Library. Terms related to study type were combined with subject-specific search terms developed through reviewing previously published systematic reviews, reviewing keywords in relevant citations, and extraction from the inclusion criteria listed in the previous subsection. Although settings and interfaces varied between these databases, a similar search strategy was adapted for each database. The relevant search terms and search filters that varied for each database were used accordingly. Subject heading indexing (eg, Medical Subject Heading terms) or the “explode” qualifier were used to expand the search language whenever possible. If no indexing was possible through subject headings, individualized search terms and more-complex strings were used through controlled language using operators and truncation. For instance, if indexing was not possible for JIA terms, proximity searching using items for “juvenile” and “arthritis” were implemented (ie, “juvenile W/3 arthritis” for Scopus or “juvenile NEAR/3 arthritis” for Web of Science). The complete search strategy used for this systematic review is available in Table S1. Search terms were organized into 2 concepts: vitamin D and JIA. Terms within each category were separated by the Boolean operator “OR,” and each category was separated by the operator “AND.” A librarian was consulted to refine each search strategy according to each database.

### Data Collection and Analysis

Covidence systematic review software (2023)^14^ was used to manage the screening of articles through the comparison of identified studies. Microsoft Excel (Microsoft Corp)^15^ was used to organize the data extraction form, which included outcomes and results. Other relevant details from each article, including but not limited to location and author information, were collected and included in the data extraction form (Tables S2 and S3).

#### Inclusion Procedure

Results of the searches were independently reviewed by 5 reviewers (R.B., K.J.B., K.H.L., E.X., K.Z) through title and abstract screening. Each study was reviewed twice. The included studies then proceeded to full-text screening, conducted by 2 of 5 reviewers simultaneously. Reasons for study exclusion were also documented. Disagreements were resolved by discussion and consensus with a third reviewer who had not previously reviewed the results of that search. Inter-rater reliability was calculated using raw percentages of agreement and the Cohen’s κ coefficient for title/abstract, and full-text screening.

#### Data Extraction

Data were extracted by 5 reviewers independently. Two reviewers were assigned to each study, and disagreements were resolved via discussion with a third reviewer. In the instance where a consensus could not be reached by a third reviewer, a meeting with the group of 5 reviewers was convened, in which the discussion of a resolution or compromise was facilitated. Any reviewers who disagreed could then present their perspectives with the opportunity for an open dialogue to consider alternative viewpoints and their implications on the interpretation. Relevant literature was consulted for any guidance on the standard methodologies or best practices in the identified scenarios. For instance, the decision to only document results for the precursor of vitamin D [25(OH)D] rather than its active form [calcitriol, 1,25([OH)2D] was agreed upon because 25(OH)D is a reliable marker of overall vitamin D status.^16^ A third party arbitrator (M.S.M.-M.) was consulted if further mediation was needed. For instance, it was agreed upon that subgroup analysis by JIA subtype was not feasible, as not all articles stratified by subtype, and among those that did, there were varying levels of subtypes reported.

The data extraction form organized using Microsoft Excel included the following characteristics: study number, first author name, year of publication, country, title, objective, duration of follow-up, study design, data source, sample size, sampling method, population, how JIA was diagnosed, and how vitamin D levels were tested. Study outcomes and results were recorded on another Excel sheet, which included the following additional characteristics: statistical model, type of measure of association, coefficient and CI quantifying the relationship between JIA and vitamin D, other considered predictors, and conclusion.

#### Data Analysis

Data from the eligible articles were first analyzed qualitatively by focusing on and comparing each reported mean of serum vitamin D level and their corresponding SD between patients with JIA and their respective control groups. Subsequently, a meta-analysis using Stata, version 4 (StataCorp) was performed using studies that reported mean serum vitamin D levels ± SD of both patients with JIA and healthy control participants. A forest plot was used to express the standardized mean differences of serum vitamin D levels between patients with JIA and the control group, with 95% confidence intervals (CIs). Studies without the correct units of interest, different comparison groups, or incorrect parameters of interest were included in the qualitative analysis.

Heterogeneity between study findings was evaluated using the *I*^2^ statistic and a forest plot was generated to visualize potential differences in effect sizes. If the *I*^2^ value was large (>50%), indicating high heterogeneity, a random-effects model would be pursued; otherwise, meta-analysis would be conducted with a fixed-effects model. A high *I*^2^ value is evidence that effect sizes between studies differ due to both random error and true variability. Studies with smaller variability and larger sample sizes were assigned a higher weight for the effect-size calculation. Although studies lacking the parameter of interest were excluded from the meta-analysis, additional subgroup analysis was not conducted due to a lack of sufficient studies.

#### Assessment of Risk of Bias

The risk of bias was assessed by 5 reviewers separately and independently, with 2 reviewers per study (differentiated as “a” and “b” in Table S2). The criteria were based on JBI critical appraisal tools^17^ and split by study design (eg, case-control studies, cross-sectional studies, cohort studies). The criteria are listed in the Supplementary Materials. The studies were evaluated on each criterion and assigned a status of high, moderate, or low concern. Articles with one or two “high” or “some concerns” ratings were classified as low risk of bias, three as moderate, and more than three as high risk. However, categories of higher importance, such as studies that did not consider confounding, were classified qualitatively instead of quantitatively. Disagreements were only resolved by discussion with a third reviewer if they were highly contrasting (eg, high vs low concern).

#### Assessment of Certainty

The Grading of Recommendations, Assessment, Development, and Evaluations (GRADE) approach was used to assess the certainty of the body of evidence. The framework is underpinned by 7 considerations: study design, with a higher quality of evidence being assigned to randomized trials as opposed to observational studies; risk of bias (as previously assessed using JBI tools); inconsistency, defined by variation in effect sizes, CIs, statistical significance, and heterogeneity between studies; indirectness, which refers to different comparisons in PICO between studies; imprecision, defined by small sample sizes and wide CIs; publication bias; and other considerations, which include factors that may raise the quality of evidence, such as the presence of a clear dose-response gradient, a very large effect (relative risk >2), or an effect of plausible residual confounding.

One reviewer examined each criterion for GRADE, not including the previous risk-of-bias assessment. The overall GRADE rating was established after a thorough discussion among all group members on the optimal grading for each category, and all group members came to a consensus based on what was deemed as an acceptable reflection of the studies’ evaluation. Additionally, a funnel plot was used to assess any publication bias due to missing or excluded results.

## RESULTS

### Search Findings and Selected Studies

**Figure 1.**
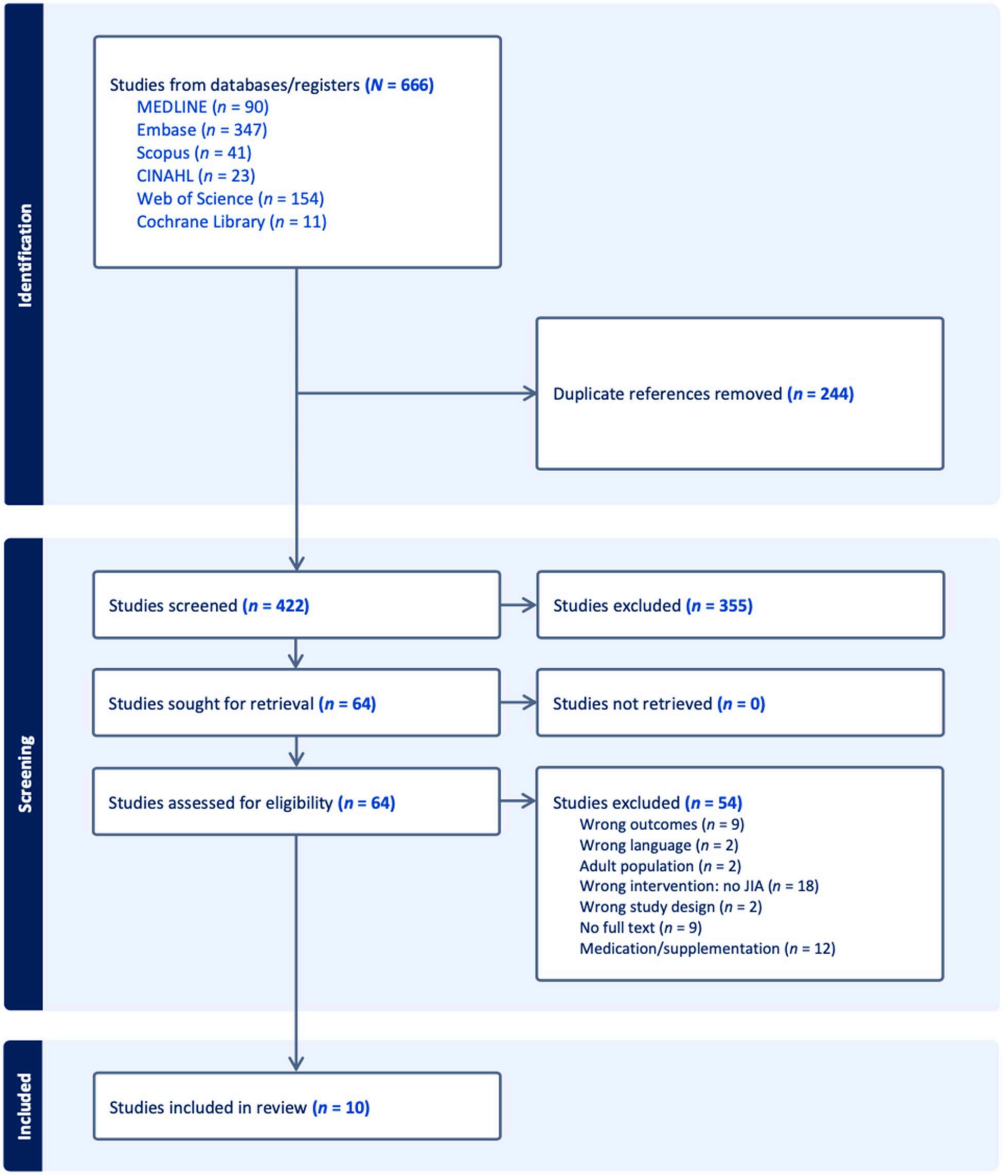
Preferred Items for Systematic Reviews and Meta-Analyses Flow Diagram

A total of 666 articles were initially identified in the literature search, of which 10 unique eligible studies were identified for inclusion in the final review (Figure 1). MEDLINE had a total of 90 applicable articles, Embase had 347, Scopus had 41, CINAHL had 23, Web of Science had 154, and the Cochrane Library had 11 (Figure 1). Of these, 244 were duplicate publications. The remaining 422 articles then went through title and abstract screening conducted by 2 of 5 reviewers based on the initial inclusion and exclusion criteria. Inter-rater reliability information for each of the 2 reviewers for title and abstract screening as well as full-text screening is available in Tables S7 and S8. All disagreements were resolved by discussion with a third reviewer. Cohen’s κ levels were classified as follows: values <0.20 were classified as poor, 0.21-0.40 as fair, 0.41-0.60 as moderate, 0.61-0.80 as good, and ≥0.8 as very good. Most disagreements stemmed from the uncertainty of including studies, which resulted in many “maybe” decisions contrasting against the second reviewer’s decision. For example, a study’s relevance came into question when it examined a transcriptional path for vitamin D receptors in JIA pathogenesis, thereby inferring vitamin D’s association with the development of JIA. Shortly after the discussion about the relevance of these studies, reviewers clarified existing ambiguity, and agreements were reached.

The authors agreed that studies examining specific biological mechanisms among other risk factors as primary outcomes should be excluded. The 3 primary reasons for excluding studies were (1) the article focused on vitamin D supplementation as an intervention; (2) the ages of participants exceeded 16 years, and stratified analyses with the subset of patients younger than 16 years were not performed; or (3) other medications were used as an intervention, such as corticosteroids or methotrexate therapy, and were not adjusted for in statistical analysis. The last reason was applied because certain medications can potentially confound results by influencing vitamin D levels if left unaccounted for, and this review aimed to examine baseline differences between patients with JIA and control participants.

Initially, certain studies may have seemed to adhere to the inclusion and exclusion criteria after full-text screening, yet upon further investigation, they did not stratify certain characteristics sufficiently to be included in the quantitative meta-analysis. However, these studies were still included in a qualitative analysis, along with the other studies, because they reported overall mean differences in serum vitamin D levels between a JIA group and a control group. Two studies^8^^,^^18^ did not have the parameter of interest of mean ± SD, using median and interquartile range (IQR) instead, or the correct units of interest (nmol/L rather than ng/mL). Therefore, these two articles were excluded from the meta-analysis but were examined qualitatively. To clarify, unit conversions were not sought for these studies for meta-analysis because, along with having the incorrect units, they only reported median (IQR). Although Finch et al^8^ did report mean (SD), they used IQR for their calculations because their data was skewed. Hence, further conversions of SD in nmol/L to ng/mL could not be pursued, owing to the skewness. The Finch et al article^8^ was also excluded because the authors used age subgroups (ages 6-16 and 3-5 years) of patients with JIA. Although Dağdeviren-Çakır et al^19^ also subgrouped their patients with JIA (by remission and activation periods), their findings for the activation period were included in the meta-analysis because the subgroups were based within the same patients with JIA, and the mean vitamin D levels did not differ statistically with the remission group (95% CIs overlapped). Although patients with JIA in the Dağdeviren-Çakır et al^19^ study also followed exclusionary drug regimens (see exclusion criteria), the drug regimens did not differ between the activation and remission periods, nor were they found to have a statistically significant correlation with vitamin D levels. Likewise, patients with JIA in the Munekata et al study^20^ followed drug regimens, but there was no statistically significant association with vitamin D levels.

### Study Characteristics

Characteristics of study participants from each of the 10 studies are shown in Table S2. Included studies had a combined total of 5542 study participants, consisting of those diagnosed with JIA before the age of 16 years and studies that included healthy children as control participants. The total systematic review consisted of 4 case-control studies, 4 cross-sectional studies, and 2 cohort studies. The geographic location of the studies varied greatly, with representation from Africa, Asia, North and South America, and Europe.

### Risk of Bias

After screening and exclusion, the only studies that met inclusion criteria were those with an observational study design. The risk-of-bias assessments are summarized separately for case-control studies, cross-sectional studies, and cohort studies, following the JBI criteria that differ for each study design (Tables S4-S6). No drastic disagreements were observed: no reviewers determined studies to be highly contrasting as simultaneously having low and high risk of bias. The overall risk-of-bias scores were averaged from the individual risk-of-bias assessments of 2 reviewers for each study. No study had an overall agreement of a high risk of bias. For case-control studies, JBI criteria were satisfied 90% of the time (Figure S1), combining an overall proportion of low risk from each study. In the cross-sectional risk-of-bias assessment, the JBI criteria were satisfied 74% of the time (Figure S2). The cohort studies had the lowest criteria satisfaction rate, 73% (Figure S3).

**Figure 2.**
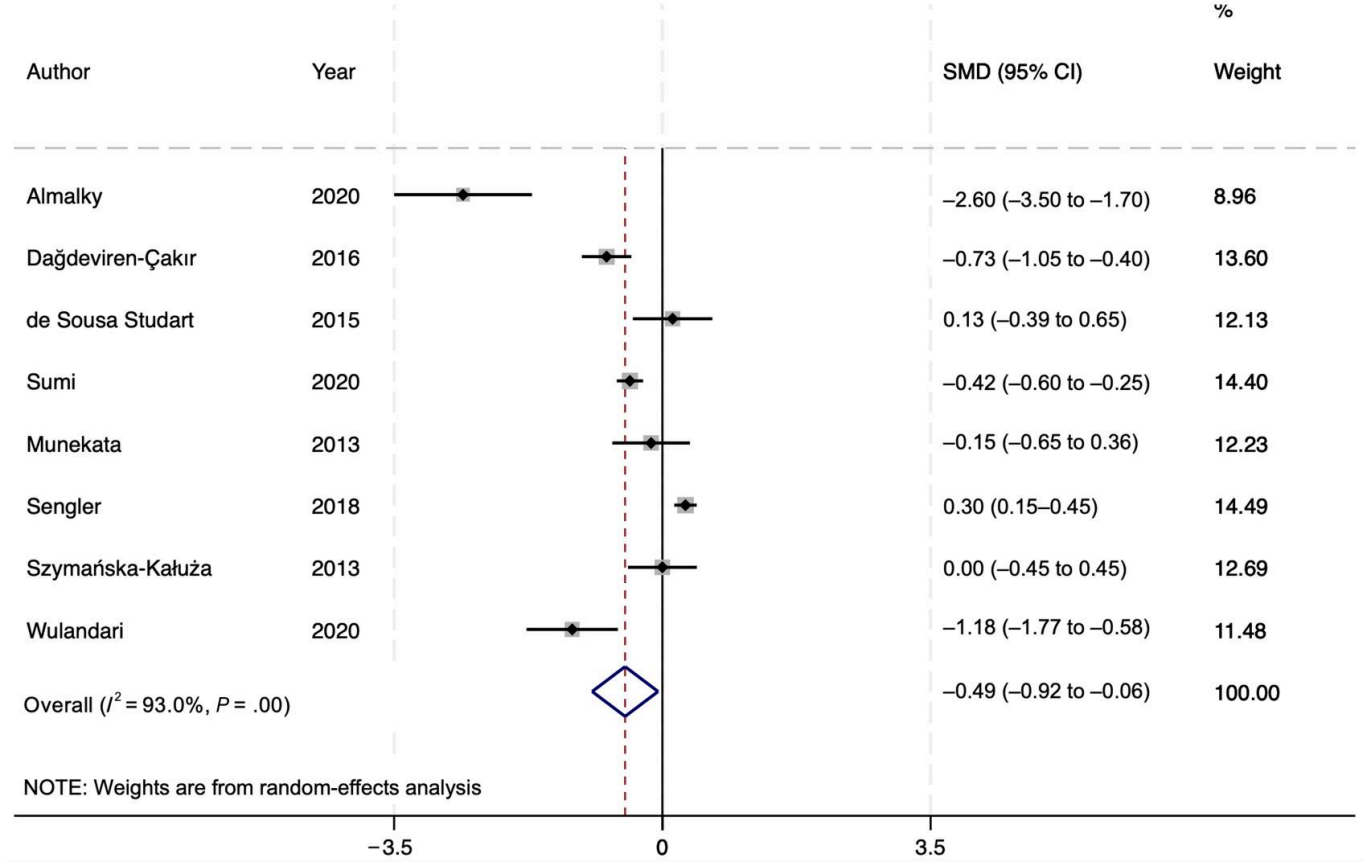
Forest Plot of the Random-Effects Model Demonstrating a Statistically Significant Effect Between the Patients with Juvenile Idiopathic Arthritis and Control Participants When Comparing Their Serum Vitamin D Levels

**Figure 3.**
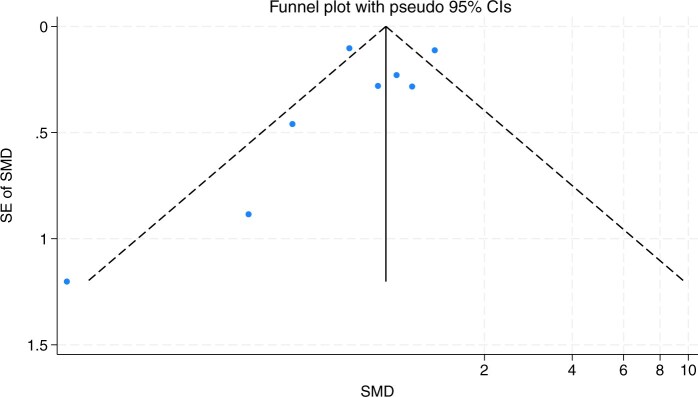
Funnel Plot of Included Studies Comparing Vitamin D Serum Levels Between Patients with Juvenile Idiopathic Arthritis and Control Participants. Abbreviation: SMD, standardized mean difference.

### Qualitative and Descriptive Findings

A total of 10 of 422 studies were eligible for qualitative analysis. Seven studies found a statistically significant difference in mean vitamin D levels between healthy control participants and patients with JIA. Within these seven, five reported a lower mean serum vitamin D level in patients with JIA as compared with their healthy counterparts.^18^^,^^19^^,^^21–23^ Contrastingly, the remaining two^8^^,^^24^ of the seven studies with statistically significant findings found a slightly higher mean serum vitamin D level in patients with JIA as compared with control participants. Three studies found no statistically significant difference between the groups.^20^^,^^25^^,^^26^ Finch et al^8^ and Ćosićkić^18^ had opposite yet statistically significant findings: One study^18^ found that the patients with JIA had a lower mean vitamin D serum level than healthy control participants, and the other^8^ reported a higher mean vitamin D serum level in patients with JIA. However, the findings of Finch et al^8^ were statistically significant for their 6-16 years age group but not for their 3-5 years age group. Overall, these qualitative results demonstrated a generally consistent finding of vitamin D deficiency in patients with JIA.

Overall, of the studies that measured vitamin D levels in nanograms per milliliter, patients with JIA had a mean serum vitamin D level of 22.75 ng/mL, and control participants had a mean level of 26.19 ng/mL. These values are both below the ideal vitamin D levels defined for children (>30 ng/mL), and both reach levels of insufficiency. Although some individuals in the control population achieved an ideal vitamin D level, these findings may highlight the prevalence of vitamin D insufficiency or deficiency in the general population. Nonetheless, patients with JIA had a lower mean serum vitamin D level on average as compared with control participants. Further quantitative analysis was pursued to determine the exact differences.

### Meta-Analysis Findings

After screening, 8 of the 10 eligible studies for review were used in the meta-analysis.^19–26^ The eligible studies consisted of 4 cross-sectional studies, 3 case-control studies, and 1 cohort study. The subsequent random-effects model (Figure 2^19–26^) found an overall effect size (standardized mean difference) of –0.49 (95% CI, –0.92 to –0.06), suggesting the identified association between vitamin D and JIA is statistically significant. A random-effects model was used due to the high heterogeneity, as demonstrated by the *I*^2^ value of 93% (Figure 2). The sources of heterogeneity are further expanded upon in the following sections. To reiterate, subgroup differences were not explored through subsequent subgroup analysis, due to the lack of sufficient studies per category (eg, JIA subtype or age).

The effect size of –0.49 suggests that patients with JIA have lower vitamin D levels, and thus vitamin D may be a modifiable risk factor for JIA that can be targeted for therapy. More research is warranted regarding vitamin D supplementation in addition to genetic mechanisms underlying JIA.

### GRADE Assessment

To assess collected evidence and determine the level of confidence in conclusions and analyses, the GRADE framework^27^ was used manually. Each of the considerations previously iterated in the *Methods* section follow guidelines for raising and lowering quality, as outlined in the GRADE handbook, which were adhered to.^27^ The rationale for the grading is outlined next, while the assessment results are summarized in Table 2.

**Table 2.** Evidence Profile (GRADE Assessment) of Included Studies

Certainty assessment (outcome measure)	Study design	Risk of bias	Inconsistency	Indirectness	Imprecision	Publication bias	Other considerations	Overall GRADE	No. of patients: JIA group; control group
Serum 25(OH)D levels	All observational	Low-moderate	Some but not critical	Some but not critical	None	Inconclusive	Dose-response gradient	Moderate	896; 4646

Abbreviations: GRADE, Grading of Recommendations, Assessment, Development, and Evaluations; JIA, juvenile idiopathic arthritis.

JBI critical appraisal tools were used to analyze the studies’ risks of bias by their study designs and methodologies. Most studies included in this review had a low risk of bias except for some specific aspects, which downgraded the overall measure. For instance, multiple cross-sectional studies did not account for confounders such as the duration of disease. The analysis stemming from the use of the GRADE framework suggests that there are some moderate concerns for the determinants of quality. A partial reason for the low quality is that all studies were observational, which inherently begins with having a “low” rating. However, many studies found a dose-response gradient, which increases the overall quality of GRADE. The moderate grading may be a result of most included studies being exploratory and descriptive, which results in less rigorous statistical analyses.

Some, but not critical, concern was established for indirectness. Dağdeviren-Çakır et al^19^ and Szymańska-Kałuża et al^26^ had different control populations than the rest of the studies; however, with all the same intervention populations, the outcome of interest was still effectively quantified. Some concern was established for inconsistency because there was high heterogeneity between the studies, but there is a lack of critical concern owing to the ability to account for the heterogeneity: Finch et al^8^ and Sengler et al^24^ reported inconsistent results due to the opposite effects of vitamin D on JIA, as compared with the remaining studies, and two studies^8^^,^^19^ used different subgroupings of patients with JIA. Inconsistency may arise from the high prevalence of vitamin D deficiency in the general population or in geographic areas with different climates.

Publication bias was determined through the assessment of each article, as well as a funnel plot (Figure 3). The funnel plot was not completely symmetrical; however, evaluating publication bias based solely on the plot is difficult due to the limited number of studies included and the presence of high heterogeneity (as seen with the *I*^2^ value). Nevertheless, the funnel plot did show moderate symmetry: points are scattered within or near the boundaries at the top and bottom left of the plot. Moreover, funnel plot asymmetry is only one way to detect publication bias.

## DISCUSSION

The key goal of this research was to examine a possible association between vitamin D levels and JIA through a comparison of serum 25(OH)D levels in patients with JIA relative to matched control participants. This goal is consistent with the research focus of this project to elucidate possible therapeutic implications of vitamin D in the management of JIA, because lower 25(OH)D levels may be a modifiable risk factor. The sample of included eligible studies included cohort, case-control, and cross-sectional studies; randomized controlled trials were not found within the eligible studies. Nonetheless, an association could be established because all comparisons underwent tests of statistical significance.

The latest systematic review on this topic, published by Nisar et al^11^ in 2013, indicated a lack of clear evidence supporting a link between vitamin D levels and disease status in patients with JIA. In an additional scoping review in 2018, Finch et al^12^ concluded that the relationship between vitamin D status and disease activity in children with JIA is still unclear. They found a high prevalence of 25(OH)D insufficiency among children with JIA; however, the optimal vitamin D status for children with JIA is unclear. The present updated systematic review reports increased vitamin D deficiencies in patients with JIA as compared with control participants in the majority of its findings. Possible factors that may have contributed to discrepancies and heterogeneity between studies may be the location of study, whereby those closer to the equator receive more exposure to sunlight and hence may have higher levels of serum vitamin D, in addition to the commonness of vitamin D deficiency in the general population.

### Strengths

Overall, there was good conflict resolution during the study inclusion and exclusion process, because all conflicts were resolved by discussion with a third reviewer. When a conflict arose (eg, during abstract screening), sufficient reasons for decisions were provided and communicated thoroughly among team members to propose the optimal resolution such that study validity was ensured. Additionally, a comprehensive search of 6 databases was completed with help from a librarian to refine search terms for the final search strategy. A comprehensive search reduced the risk of overlooking relevant studies, and refining the strategy enhanced its completeness, reliability, and reproducibility. Moreover, the predefined inclusion and exclusion criteria were followed strictly, after resolving any preliminary misunderstandings. This strict adherence to the criteria reduced discrepancies in classifying studies.

### Limitations

There were drawbacks arising from all included studies being observational, which automatically assigned them a low quality rating according to the GRADE framework. Additionally, JIA has many subtypes, each with a different biological mechanism, making it difficult to retrieve data on each subtype. Thus, findings tended to be generalized to all subtypes, which may lower external validity through the homogenization of the disease. The subtypes could not be accounted for, due to the lack of consistent subgrouping among all studies. Nevertheless, the findings provide valuable preliminary insights into vitamin D as a modifiable risk factor for JIA, underscoring the importance of continued research efforts within this field and its diverse subtypes. Last, excluding articles involving patients taking additional medications omits many studies that may otherwise be informative, given that such treatments are commonly prescribed to patients with JIA to alleviate pain and manage symptoms. Nonetheless, exclusion of these medicated patients gave a better understanding of the baseline differences between patients with JIA and healthy individuals.

## CONCLUSION

Findings from this systematic review and meta-analysis provide insight and evidence for vitamin D being a mediating inflammatory factor affecting JIA development and possible severity. Additional research into a comparison of vitamin D levels across JIA subtypes may provide more evidence for influence on disease severity, as well as indicate further research on the efficacy of using vitamin D supplementation as therapy.

Because the etiology of JIA remains unknown, continuance of research funding to bridge the gaps in knowledge is essential. In particular, there is a need to investigate biological mechanisms and agents to improve biological therapies for JIA. Moreover, because there are many different JIA subtypes and causal mechanisms, further research is necessary to explore the differences and similarities among the subtypes to improve JIA treatment. Many medications prescribed to patients with JIA, such as glucocorticoids, can lower vitamin D levels; therefore, they should be investigated as potential mediators or confounders in the hypothetical JIA causal framework. Prospective studies of individuals before the development of JIA are needed to provide full evidence of the influence of vitamin D on JIA prevention.

## Supplementary Material

nuae148_Supplementary_Data

## Data Availability

Full data extracted from included studies, data used for all analyses, analytic code, and any other materials used in the review can be accessed through the supplemental materials, or contacting the authors.
