# Correction to: Vitamin D as a Modifiable Risk Factor for Juvenile Idiopathic Arthritis: A Systematic Review and Meta-analysis of Observational Studies Comparing Baseline Vitamin D in Children with JIA to Individuals Without

**DOI:** 10.1093/nutrit/nuae186

**Published:** 2024-11-15

This is a correction to: Kathleen Zang, Resham Bhatia, Elizabeth Xue, Kalia J Bennett, Katherine H Luo, Monali S Malvankar-Mehta, Vitamin D as a Modifiable Risk Factor for Juvenile Idiopathic Arthritis: A Systematic Review and Meta-analysis of Observational Studies Comparing Baseline Vitamin D in Children with JIA to Individuals Without, *Nutrition Reviews*, 2024; https://doi.org/10.1093/nutrit/nuae148

In the originally published version of the manuscript, an error was introduced into the title of Figure 3. This should read: “Funnel plot with pseudo 95% CIs” instead of: “Funnel plot with pesudo 95% CIs”

The emendation has been made to the article.

