# Supplementary material for: Vitamin D as a Modifiable Risk Factor for Juvenile Idiopathic Arthritis: A Systematic Review and Meta-analysis of Observational Studies Comparing Baseline Vitamin D in Children with JIA to Individuals Without
Source: Nutr Rev. 2024 Oct 25;83(7):e1362–71. doi: 10.1093/nutrit/nuae148 (PMC12166184; doi:10.1093/nutrit/nuae148)
Supplement: nuae148_Supplementary_Data [file nuae148_supplementary_data.zip › nuae148_Supplementary_Data/S2.docx]

**Table S4.**  Risk of Bias Assessment for Case-Control Studies - JBI Criteria

**
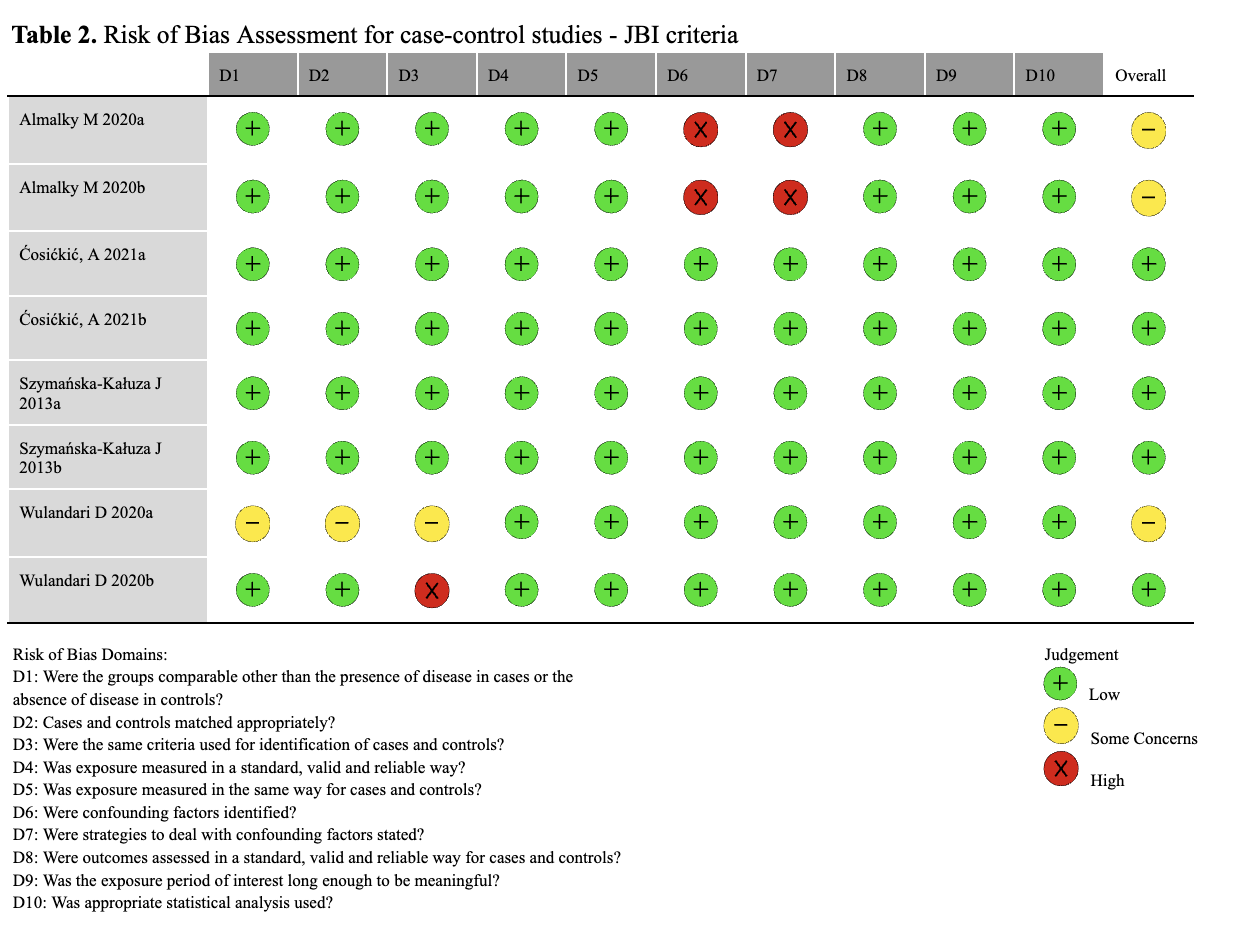
**

**Table S5.**  Risk of Bias Assessment for Cross-Sectional Studies - JBI Criteria

**
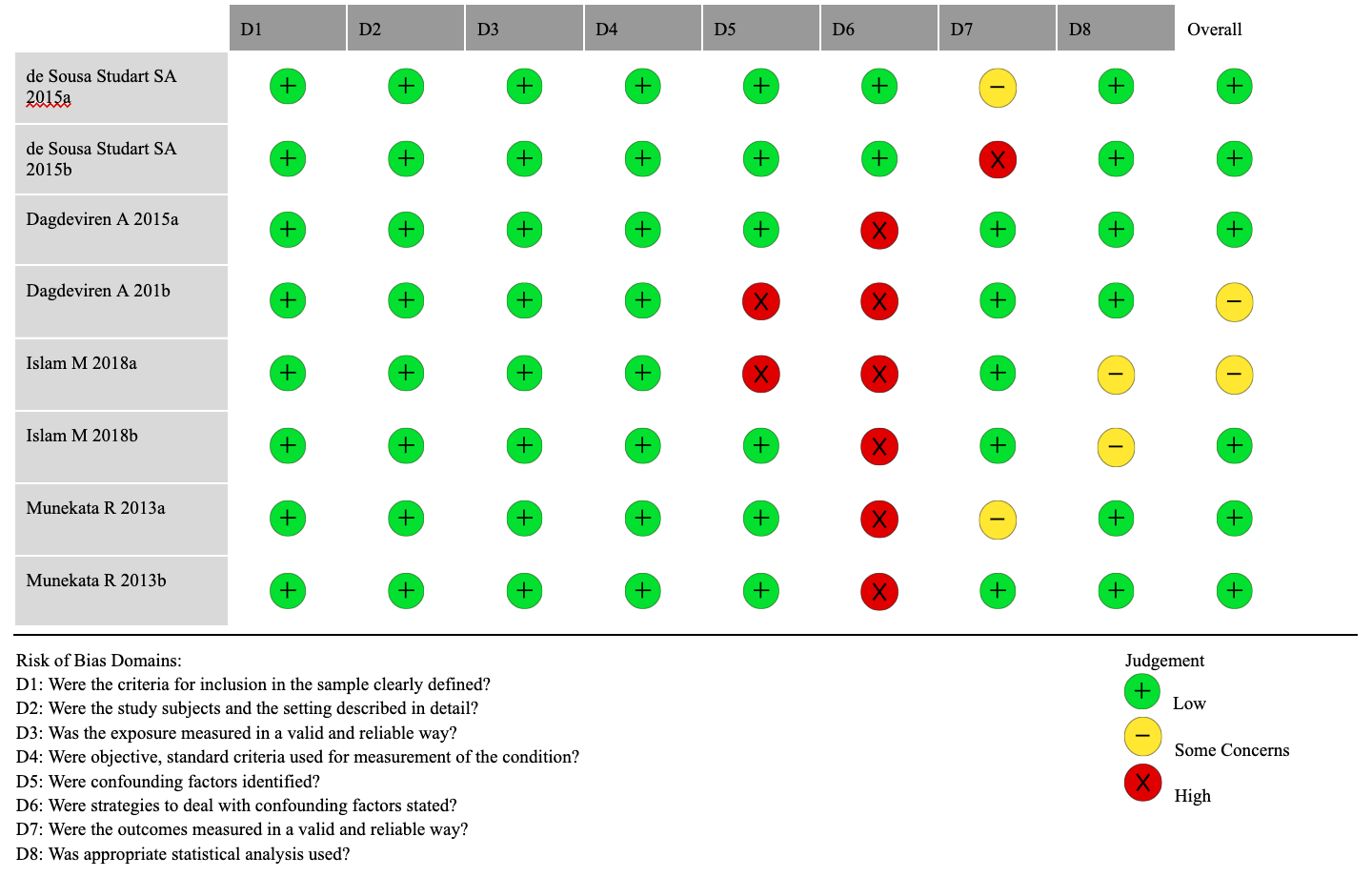
**

**Table S6.** Risk of Bias Assessment for Cohort Studies - JBI Criteria

**
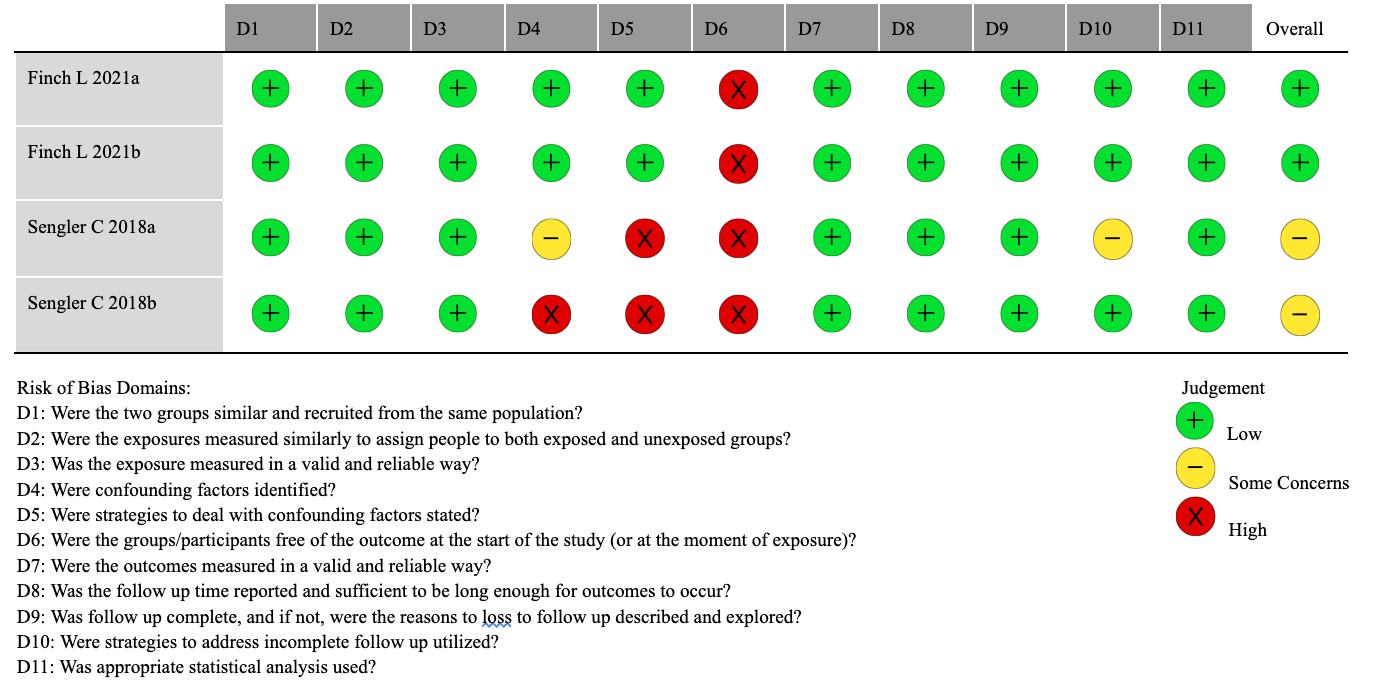
**

**Table S7.**  Inter-rater reliability for Title and Abstract.

| *Reviewer A* | *Reviewer B* | *A Include*  *B Include* | *A Include*  *B Exclude* | *A Exclude*  *B Include* | *A Exclude*  *B Exclude* | *Proportionate Agreement* | *Yes Probability* | *No Probability* | *Random Agreement Probability* | *Cohen’s Kappa* |
| --- | --- | --- | --- | --- | --- | --- | --- | --- | --- | --- |
| K.L | R.B | 4 | 0 | 3 | 14 | 0.85714 | 0.06349 | 0.53968 | 0.60317 | 0.64 |
| E.X | K.Z | 13 | 0 | 3 | 24 | 0.925 | 0.13 | 0.405 | 0.535 | 0.83871 |
| K.B | K.Z | 13 | 0 | 16 | 47 | 0.78947 | 0.06527 | 0.51264 | 0.57791 | 0.50123 |
| E.X | K.L | 4 | 0 | 0 | 14 | 1 | 0.04938 | 0.60494 | 0.65432 | 1 |
| K.Z | R.B | 5 | 1 | 2 | 31 | 0.92308 | 0.02761 | 0.69428 | 0.72189 | 0.7234 |
| E.X | K.B | 4 | 0 | 1 | 32 | 0.97297 | 0.01461 | 0.77137 | 0.78598 | 0.87372 |
| K.B | R.B | 2 | 1 | 2 | 26 | 0.90323 | 0.01249 | 0.78668 | 0.79917 | 0.51813 |
| K.L | K.Z | 5 | 4 | 2 | 21 | 0.8125 | 0.06152 | 0.56152 | 0.62305 | 0.50259 |
| E.X | R.B | 7 | 0 | 0 | 86 | 1 | 0.00567 | 0.85513 | 0.86079 | 1 |
| K/B | K.L | 7 | 3 | 0 | 26 | 0.91667 | 0.05401 | 0.58179 | 0.6358 | 0.77119 |


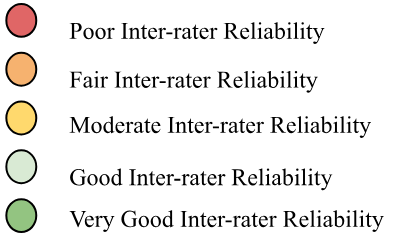


**Table S8.** Inter-rater reliability for Full Text Screening.

| Reviewer A | Reviewer B | A Include  B Include | A Include  B Exclude | A Exclude  B Include | A Exclude  B Exclude | Proportionate Agreement | Yes Probability | No Probability | Random Agreement Probability | Cohen’s Kappa |
| --- | --- | --- | --- | --- | --- | --- | --- | --- | --- | --- |
| E.X | R.B | 2 | 3 | 0 | 0 | 0.4 | 0.4 | 0 | 0.4 | 0 |
| E.X | K.Z | 7 | 4 | 0 | 3 | 0.71429 | 0.39286 | 0.10714 | 0.5 | 0.42857 |
| K.B | K.Z | 5 | 1 | 0 | 0 | 0.83333 | 0.83333 | 0 | 0.83333 | 0 |
| E.X | K.B | 0 | 3 | 0 | 1 | 0.25 | 0 | 0.25 | 0.25 | 0 |
| K.B | R.B | 3 | 2 | 1 | 3 | 0.6667 | 0.24691 | 0.24691 | 0.49383 | 0.34146 |
| K.L | K.Z | 2 | 3 | 1 | 3 | 0.55556 | 0.18519 | 0.2963 | 0.48148 | 0.14286 |
| K.Z | R.B | 3 | 1 | 0 | 2 | 0.83333 | 0.33333 | 0.16667 | 0.5 | 0.66667 |
| K.B | K.L | 1 | 1 | 0 | 2 | 0.75 | 0.125 | 0.375 | 0.5 | 0.5 |
| K.L | R.B | 0 | 0 | 1 | 0 | 0 | 0 | 0 | 0 | 0 |


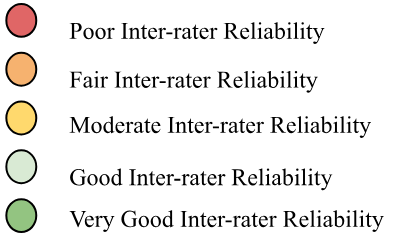


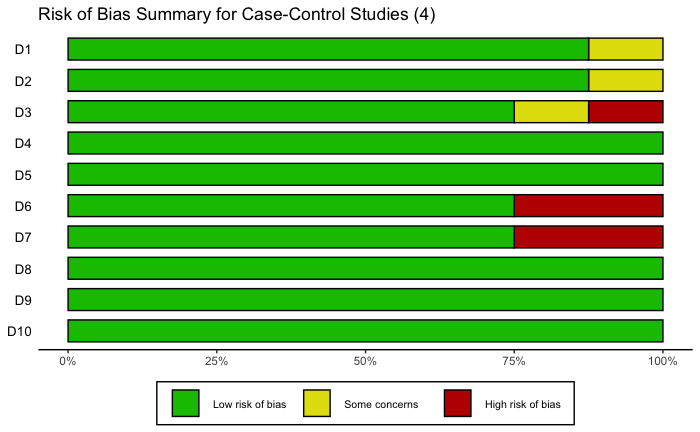


**Figure S1.** Risk of Bias Summary for Case-Control Studies.


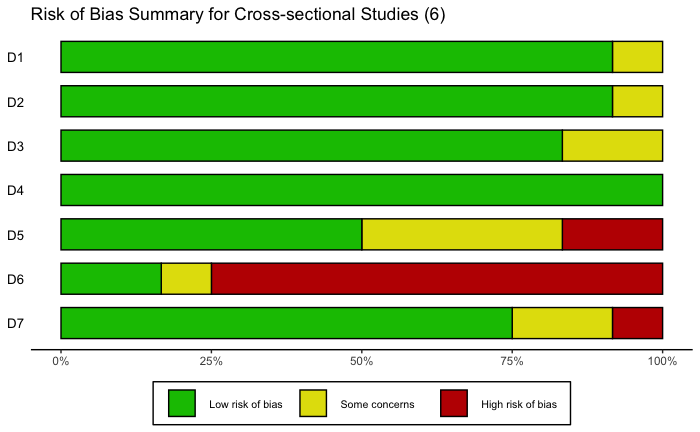


**Figure S2.** Risk of Bias Summary for Cross Sectional Studies.


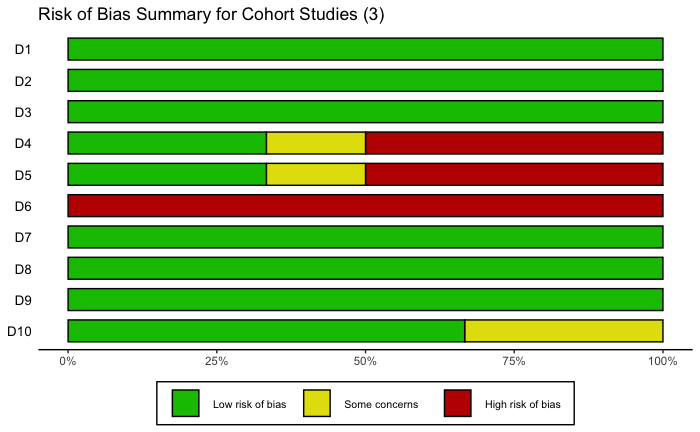


**Figure S3.** Risk of Bias Summary for Cohort Studies.
